# Bee Swarm Activity Acoustic Classification for an IoT-Based Farm Service

**DOI:** 10.3390/s20010021

**Published:** 2019-12-19

**Authors:** Andrej Zgank

**Affiliations:** Faculty of Electrical Engineering and Computer Science, University of Maribor, SI-2000 Maribor, Slovenia; andrej.zgank@um.si; Tel.: +386-2-220-7206

**Keywords:** bee acoustic analysis, activity monitoring, acoustic classification, IoT architecture

## Abstract

Beekeeping is one of the widespread and traditional fields in agriculture, where Internet of Things (IoT)-based solutions and machine learning approaches can ease and improve beehive management significantly. A particularly important activity is bee swarming. A beehive monitoring system can be applied for digital farming to alert the user via a service about the beginning of swarming, which requires a response. An IoT-based bee activity acoustic classification system is proposed in this paper. The audio data needed for acoustic training was collected from the Open Source Beehives Project. The input audio signal was converted into feature vectors, using the Mel-Frequency Cepstral Coefficients (with cepstral mean normalization) and Linear Predictive Coding. The influence of the acoustic background noise and denoising procedure was evaluated in an additional step. Different Hidden Markov Models’ and Gaussian Mixture Models’ topologies were developed for acoustic modeling, with the objective being to determine the most suitable one for the proposed IoT-based solution. The evaluation was carried out with a separate test set, in order to successfully classify sound between the normal and swarming conditions in a beehive. The evaluation results showed that good acoustic classification performance can be achieved with the proposed system.

## 1. Introduction

Digital farming is one of the key technologies which has been gaining support through different research initiatives in the last decade. The fundamental digital farming setup consists of the central management platform [1], human–computer interfaces [2] and particular IoT modules [3], which monitor the animals, their behavior and facilities. All these building blocks are connected [4] into a common platform, using communication networks which are, today, frequently based on IoT architecture combined with cloud or edge-based functionalities. The intelligent ambiance is, in a scenario of digital farming, a more sophisticated concept, which enables information processing from big data, combined with current context and conditions, and adequate adaptation according to the prior knowledge or decisions resulting from machine learning algorithms. An intelligent ambiance for farming needs to include different sensors and sensor networks. These are necessary for the system to be able to build on various information collected as part of context modeling. A broad range of information processing applications [5] and Information and Communication Technology (ICT) services can be developed and integrated as a result. Notification and alarm services, in combination with real-time monitoring (mobile) applications, are some of those important features which ease the daily operations, particularly in the case of large and distributed facilities. The main reasons responsible for this IoT success are miniaturization, the reduced energy consumption of IoT devices with prolonged autonomy and the rapid development of wireless technologies.

In the case of mobile networks, the 5G, and the already existing LTE-M1/NB-IoT, enable ubiquitous connectivity, which is important when the system gets distributed over more than one location. The increased number of connected devices, many of them originating from wireless-distributed sensor networks, were also responsible for the last major changes in network protocol standardization, where IPv6 was defined and deployed to communication networks, with the objective to cope with the new situation.

If IoT and intelligent ambiance present one of the upfront sectors, agriculture is still a more traditional one. ICT technologies were introduced widely [6] to agriculture in the last two decades, but there is a large area of possibilities on how, additionally, to include real-time monitoring, machine learning and sensor networks into this sector. Sensor networks enable numerous solutions for smart farming [7], intending to increase the production rate and improve sustainability, but they are also, in parallel, in charge of taking care of animal condition and health. Beekeeping is an important part of agriculture, where the distributed locations are frequently present, and thus, indicate the need to ease the monitoring of animals in a 24/7 mode, which can benefit from advanced intelligent ambiance technologies. The popularity of modern urban beekeeping initiatives has gained further momentum to this research field, and presents an additional possibility as to how to include digital agriculture into a smart city and smart home solutions [8].

Swarming has always been seen by beekeepers as an extremely important event, which requires an immediate response. Beekeepers were used to observing the beehive visually to detect swarming. When the beehives were distributed over a larger area, this resulted in increased costs associated with transportation and accessibility. Swarming occurs when specific conditions are met in the beehive. The old queen bee is confronted with a new queen bee, which takes over the hive. The old queen, accompanied by part of the worker bees, leaves the hive and starts a new colony in a new hive. These specific hive conditions can be monitored with an IoT-based module, and the processed information can be used for alarming the beekeeper via a digital farm alarming service. Furthermore, this solution can be integrated into a smart farming system, to establish a complete overview of animals and their conditions.

The presented paper has three main objectives. The first one is to propose a concept of an IoT-based bee swarm monitoring system, which can operate with audio signals captured in a beehive. Swarming bees generate a specific audio signal, which can be classified with machine learning approaches. The second objective is to define the most suitable classification configuration for the acoustic monitoring of a beehive, which results from the proposed IoT-based architecture. The basic concept, how to detect swarming from the audio signal, was presented by Zgank [9]. The approach proved the applicability of the experimental setup and methods used, but the results showed that further attention should be given to acoustic modeling to improve the results. Thus, a system with Gaussian Mixture Models (GMM) and Hidden Markov Models (HMM) with different topologies is proposed to analyze the swarm classification performance. Additionally, two different feature extraction procedures and denoising of acoustic background were included to increase robustness. The third objective is to raise awareness about the possibility of using openly available data for developing artificial intelligence (AI) solutions in the digital farming domain. The open data availability has already proved its success in the area of human–computer interaction, where a significant boost of research activities and gain in performance has resulted from freely-available data [10,11].

The paper is organized as follows: The review of relevant literature is given in Section 2. The materials and methods are presented in Section 3, where subsections on bee sound analysis are described, an openly available dataset that was used as audio material for the experiments and the experimental setup. The acoustic classification results obtained on the test set are reported in Section 4. The conclusion is given in Section 5.

## 2. Related Work

The topic of bee swarm acoustic activity detection using an intelligent ambiance system addresses interdisciplinary knowledge. There were already in 1950 the first attempts to analyze bees’ sounds with simple approaches [12]. New solutions were proposed with ICT technology development. The analysis of acoustic signals produced by bees was carried out by [13,14,15], with the resulting definitions of frequency bands where the sound energy is concentrated.

The next step was to define the appropriate feature extraction algorithm, which can be used thereafter for the classification task. The authors in [16] used spectrograms, FFT and S-transform to carry out an acoustic analysis. Qandour et al. [17] included the following feature extraction methods: Peak frequency, spectral centroid, bandwidth and root variance frequency. The Linear Predictive Coding (LPC), which is also used frequently in the case of human interactive systems, was applied as a feature extracting procedure in [18].

Machine learning algorithms can be used for pattern recognition and classification in the case of bee activities’ acoustic monitoring. The Kohonen Self-Organizing Map (SOM) artificial neural networks (ANNs) were applied successfully for the classification task in [16]. Machine learning approaches, such as Support Vector Machine (SVM) and Linear Discriminant Analysis (LDA) were incorporated by Qandour et al. [17] for acoustic monitoring of the animal activities in a beehive, with comparable results. Several deep learning algorithms were compared in [19] with the standard machine learning approaches, with promising results for real-life applications. In [20], the authors compared SVM and Convolutional Neural Networks (CNNs) for recognizing the beehive status from audio signals. The authors reported that the SVM approach provided a better generalization effect over the CNN. Zacepins et al. [7], Gil-Lebrero et al. [21] and Zacepins et al. [22] proposed various acoustic monitoring systems for bees. The research area still has open questions [8], particularly if IoT architecture, accuracy and overall context questions are also considered.

Besides the acoustic monitoring, the swarming activity can be detected by monitoring the environmental parameters of a beehive [8,23]. Some monitored parameters are hive temperature and humidity. This information can be used, isolated or in a data fusion mode with other data sources, such as audio classification. The goal of the data fusion mode is to model a broader context, which should lead to improved classification results.

## 3. Materials and Methods

**Figure 1:**
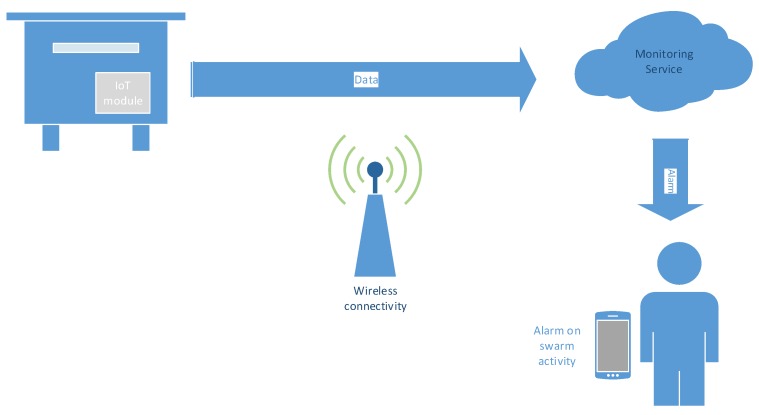
IoT-based bee swarm activity monitoring service.

The IoT-based concept of the bee swarm activity acoustic classification system is given in Figure 1.

There are two main architecture possibilities of how an IoT-based monitoring service can be designed. The crucial point is the IoT module and its properties, namely, processing power and memory. In the case when the IoT module has low processing power and memory, its only function is to capture the audio signal in the beehive and transmit it over a wireless connection to a backend server in a cloud (or on the edge), where acoustic classification takes part. When swarm activity is detected, the beekeeper is alarmed via the service, preferably on a smartphone or wearable device. A variant of such a simple IoT module can carry out feature extraction (see Section 3.3.1) and transmit feature vectors instead of audio signals. This distributed solution reduces the necessary wireless bandwidth, but increases the computational power needed on the IoT module. Thus, it applies in situations with limited wireless connectivity.

The opposite design approach is when the IoT module has enough processing power and memory to run acoustic classification locally in the beehive. Here, only the data about the occurring activity is transmitted to the monitoring service, which then alarms the beekeeper. Thus, the amount of transmitted data is as small as possible. The two proposed IoT-based concepts differ from the point of view of how complex processing takes place in the beehive. This issue will be addressed with different models’ topologies and complexities during the acoustic training.

### 3.1. Bee Behavior from an Acoustic Perspective

The captured beehive audio data contain different bees’ activities, which were studied in detail by behavioral science [12]. Four types of sounds are connected with daily activities in a beehive. The main characteristics that mark each of the categories are the frequency and energy of the signal. (1) Flying produces sound around 250 Hz. Young bees have different wing properties and produce a sound of higher frequency. (2) Pipping is the sound indicating a possible swarm event. It is a challenge signal produced by a queen bee for any potential new queen bee. The sound’s frequency range is between 340 Hz and 450 Hz. (3) A hissing sound, with a frequency around 3000 Hz, results from a defensive reaction when an intruder approaches the colony, and is produced by worker bees. (4) Fanning is produced when worker (female) bees are trying to ventilate the beehive due to worse environmental conditions, mainly temperature. Its frequency range is 225 Hz–285 Hz, and overlaps with the flying sound. The hissing and fanning sounds can also be a potential signal for a forthcoming swarm activity.

The bees’ activity acoustic monitoring solution presented in this paper combines all swarm activity indicating sounds into a straightforward solution, where only two classification categories were included in the design. The first category was the normal activity; the second one was swarm activity, despite the original subcategory to which the recording belonged. This design simplification reduces the system’s complexity, which is important with IoT-based solutions.

### 3.2. Open Audio Data

The intelligent ambiance systems, and particularly, the machine learning approaches, are based on the availability of a large amount of data. The area of spoken language technologies has already shown that openly accessible data contribute significantly to the research work in general. To extend this policy to the area of digital agriculture, the open audio data recorded as part of the Open Source Beehives Project [23] were used for the proposed system. The goal of the Open Source Beehives Project (OSBP) is to promote ICT technology in the beekeeping domain. The OSBP started as a citizen science initiative, connecting amateur members from different countries who had access to beehives. In the initial phase, various single board computers and microphones were applied to capture the beehive data. The OSBP authors [24] have, in the second phase, designed an Atmel ATmega32U4-based beehive hardware module, equipped with various sensors to collect the environmental data in a beehive. The module measures the following parameters: temperature, humidity, and barometric pressure. A TDK InvenSense ICS-40300 microphone (MEMS, Omnidirectional, −45 dB; 6 Hz~20 kHz; manufacturer: TDK InvenSense, San Jose, CA, USA) is also placed on the module board, which captures the sounds in the beehive.

The collected data were either stored locally or transmitted over the IEEE 802.11 Wi-Fi [25] or a mobile network. The captured data were stored on OSBP cloud storage, where they are available to the community. These OSBP recordings were a primary source of data for our experiments. The OSBP authors collaborated with various beekeepers around the world, where the data were captured. The OSBP data procedure combines different hardware setups. The equipment position and orientation inside the beehive can vary between locations. In addition, different types of beehives were involved in the OSBP data collection task. Thus, we have selected recordings from a single beehive as a data source, to avoid the influence of various recording conditions on the classification procedure.

The preliminary study [9] used only a limited amount of training material to prove the basic principles. The proposed study of acoustic models’ topologies and improved feature extraction procedures needed a larger quantity of audio material. Therefore, the data were extended with additional recordings, which were available from the OSPB project. The initial set had 15 normal activity recordings from a single beehive; each was 15 min long. Three swarm activity recordings were used for the second classification category; again, they were 15 min each. The number of normal activity recordings was shortened randomly, to have the same amount of audio for both classes. This step was necessary to avoid the overfitting of a single class during the training procedure. The total duration of the training set was 90 min. The audio files were annotated with the beehive condition labels, originating from the OSPB data. For the evaluation, a separate test set was constructed from a different set of OSPB audio recordings. The duration of the test set was 32 min. The original recordings were segmented into 3-s homogenous parts needed for feature extraction. The training set had 1800 recordings, and the test set had 643 recordings. The captured audio was down-sampled to a 16 kHz sampling rate, 16-bit resolution, mono channel. Each processed audio part was also accompanied by the activity label that was needed for the acoustic models’ training.

Beehives can be placed in various geographic locations with a different acoustic background. In the case of a rural environment, the level of background noise can be minimal. Moreover, the audio capturing device is usually positioned inside the beehive, which also reduces the influence of acoustic background on the audio signal. On the other hand, with urban beekeeping, the acoustic background can present an important degradation factor. This parameter was simulated with a city street ambiance noise recording, which was added to original audio data for the last part of the evaluation. The noisy test set was prepared in two versions, one with low-level noise (SNR 42.74) and one with high-level noise (SNR 27.30). All other properties of this augmented noisy test set were identical to the primary test set.

### 3.3. Development and Acoustic Classification Setup

**Figure 2:**
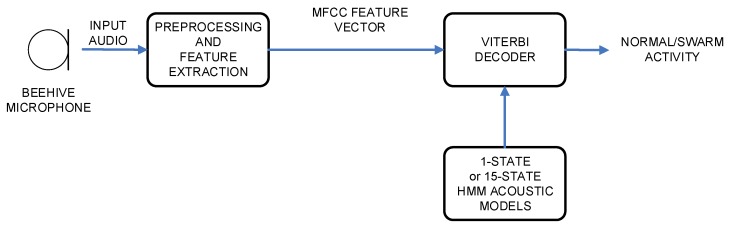
Bee activity acoustic classification system’s block scheme using Hidden Markov Model (HMM) acoustic models.

**Figure 3:**
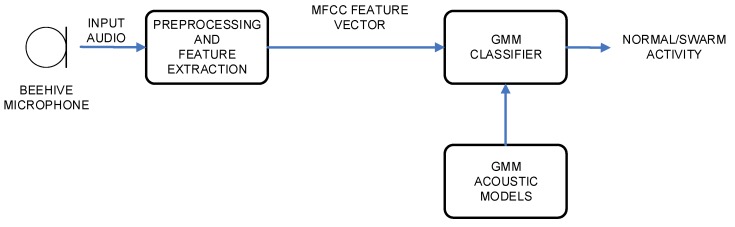
Bee activity acoustic classification system’s block scheme using Gaussian Mixture Model (GMM) acoustic models.

The experimental setup was constructed in a way to include architectures and algorithms, which originate from the areas of speech recognition and speech classification [26,27]. Thus, two different architectures were part of the design. The first one was based on Hidden Markov Models, and the second one was based on Gaussian Mixture Models. The block scheme of HMM architecture is given in Figure 2, and the block scheme of GMM architecture is given in Figure 3.

Both architectures were using an identical first part, which carried out the audio signal preprocessing and feature extraction. The second part performed the actual classification (i.e., HMM or GMM) of the data, and resulted in the final decision on the bees’ activity. All modules will be described in the following subsections.

The first step needed to perform our acoustic classification was focused on feature extraction, where the input digital acoustic signal *s_i_* was converted into a feature vector. The Mel-Frequency Cepstral Coefficients (MFCC) and Linear Predictive Coding (LPC) were selected for this task.

#### 3.3.1. MFCC Feature Extraction

**Figure 4:**
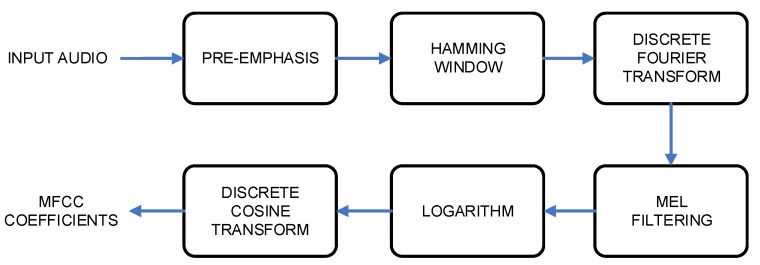
Mel-Frequency Cepstral Coefficients (MFCC) feature extraction block scheme.

The block scheme of Mel-Frequency Cepstral Coefficients feature extraction, which is also used frequently for human speech recognition, is presented in Figure 4.

**Figure 5:**
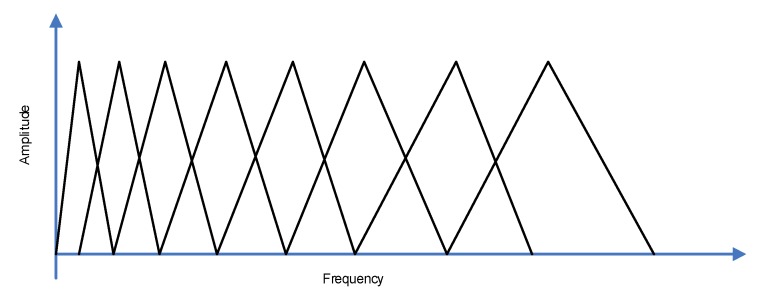
Mel-scale filter bank with eight filters.

The input audio signal was first preprocessed, which started with the preemphasizing approach to flatten the input signal spectrally. The acoustic modeling procedure needed a signal which was stationary in time, thus, framing was applied. Each frame had 25 ms’ length, and was shifted by 10 ms. The Hamming window was applied to the frames. The signal energy in each frequency band was calculated applying the Mel-scale filter bank (see Figure 5), which, typically, includes 20–30 triangular filters of an audio signal.

The last step of the MFCC feature extraction procedure was to include a Discrete Cosine Transform, which was applied to convert the log Mel spectrum into the time domain, and in parallel, to reduce the degree of correlation.

The included acoustic feature extraction generated 12 Mel-Frequency Cepstral Coefficients. An energy coefficient was added, which is standard practice for similar audio/speech classification tasks [28]. The first- and second-order derivatives were calculated and added to the primary 13 elements which resulted from the MFCC algorithm. This additional feature extraction step improved the robustness of the acoustic modeling approach, but increased the complexity of the system. The features used for the acoustic modeling training procedure had, at the end, 39 coefficients, which is comparable with speech-based systems.

The second, separate, set of feature vectors was again generated with the MFCC algorithm, but, this time, the cepstral mean normalization was applied. The normalization is a frequently used approach, which improves the robustness of the feature extraction procedure, particularly to changing conditions in a recording environment. This could be important, as recordings captured over the longtime period were also part of the audio database.

#### 3.3.2. LPC Feature Extraction and De-Noising

The Linear Predictive Coding was used for comparison as the second feature extraction method. The LPC features are similar to the MFCC features also used frequently in the area of human spoken language technologies. The input audio signal was first preprocessed and windowed using the Hamming window. The error signal (residual error) was defined between the original and its model’s representation. The signal was predicted from previous values by a weighted sum, which is called a linear predictor. The resulting system of equations can be solved efficiently with Durbin recursion.

Spectral subtraction was included as an additional preprocessing module for the evaluation of background noise. It was only applied to the noisy test set. The objective was to reduce the additive background noise, which was used to simulate the location of a beehive in an acoustically adverse environment. Spectral subtraction functions in the frequency domain, where the spectrum of a noisy signal is computed with Fast Fourier Transform (FFT) [29]. The average magnitude of this signal is calculated and subtracted from the audio input signal. The resulting signal has a lower level of background noise, and can be used for feature extraction.

#### 3.3.3. HMM Acoustic Modeling

Two different acoustic models’ topologies were included in the training process in the case of the HMM approach [30]. The HMM topology, which originated directly from automatic speech recognition, had fifteen states, and as such, was comparable with word acoustic models deployed for speech recognition. The second HMM topology had only a 1-state acoustic model. This acoustic model topology was basically identical to the acoustic models in the case of the Gaussian Mixtures Models’ classifier, with a similar number of models’ parameters. Density, mean, variance and weight of the Probability Density Function (PDF) were estimated per each model’s state. The 1-state HMM acoustic models and GMM acoustic models are similar from the perspective of the data they model, but the important difference is in the procedure of how the final decision is produced. When HMMs are used, the decoder already outputs the result. On the other hand, with GMM acoustic models, an additional step is needed, where frame occurrences are calculated. Also, a greater part of the HMM-based system is similar to human speech recognition systems. This is especially welcome when the beehive monitoring works as an IoT-based module for digital farming, as parts of the system could be reused.

The HMM acoustic modeling training procedure followed the common recipe for both acoustic model topology types. The left–right continuous density Hidden Markov Models with one Gaussian probability density function per state were chosen for the initial acoustic models. Manual check-up of audio data revealed that part of the recordings also included silence. Therefore, a special silence model with a left–right topology was also added to the setup. This modeled the silence part of the signal explicitly. Thus, the acoustic model set included silence, normal and swarm activity. Similar end results could be also achieved if the signal preprocessing stage would be enhanced with a sound activity detection algorithm, which would exclude silence from training. Here, only two acoustic models would be needed. The disadvantage of sound activity detection is the increased complexity of an additional algorithm.

The training process was divided into two parts, as it was necessary to improve the audio labels, which originated from the OSPB open audio database. The acoustic training started on the full training set with 1800 balanced recordings. First, the acoustic models’ parameters were initialized. The flat-start approach was used for the parameters’ initialization, where identical global values were assigned to all three models. Two iterations of the Baum–Welch reestimation algorithm were applied to acoustic models to train the models’ parameters. Afterward, the number of mixtures of Gaussian probability density functions was increased by a factor of two. This stepwise procedure was repeated to prevent drastic changes of values. The acoustic models in the first stage were trained until reaching 16 Gaussian mixtures per state. This was sufficient to carry out forced realigning, where training labels were matched to acoustic data. As a result of realigning, the training set contained 1.4% of outliers for 1-state acoustic models, and 1.2% of outliers for 15-state acoustic models. The outliers were excluded from the forthcoming training set, which was done for each type of HMM acoustic model separately.

The HMM acoustic models’ training continued with the second stage, where the parameter values of each acoustic model were first initialized with the local values of each class. Thereafter, the acoustic models’ training procedure started with the Baum–Welch algorithm, where three training iterations were carried out. After each training cycle, the number of Gaussian probability density functions was increased by a factor of two. The stepwise training procedure ended when 32 Gaussian mixtures per state were achieved. The final acoustic models of different types and topologies were then used for the acoustic classification task on the test set data.

#### 3.3.4. GMM Acoustic Modeling

The GMM training procedure was simpler compared to the HMM training procedure, as only one step of model training was applied, with no improvement of the labels’ quality using the forced realigning procedure. The training of the GMM acoustic models started with flat-start parameter initialization, where global values were estimated. The acoustic model training was continued with the Baum–Welch reestimation, which was repeated twice per each training cycle. Afterwards, the number of mixtures of Gaussian probability density functions was increased by a factor of two. The stepwise procedure combining training with Baum–Welch reestimation and increasing the number of mixtures, was repeated until 32 Gaussian PDF mixtures per state were reached. It would be possible to increase the number of Gaussian mixtures per state even further if more acoustic training material would be available, although it could not be guaranteed that the increased amount of training material would always improve the audio classification accuracy.

The final GMM acoustic models were included in the GMM classifier, which was fed with the input feature vectors. The GMM classification was carried out on the frame level. The final decision about the class of input acoustic signal resulted from the majority vote, which took place in the post-processing algorithm.

In comparison with the HMM procedure, the GMM procedure presented a less complex task, but the necessary GMM classification modules would not always be available in human–computer interfaces of an intelligent ambiance. Therefore, the final design decision should be a compromise between the availability of digital signal processing modules and the complexity of the algorithms.

The complexity and number of acoustic models’ parameters are usually reflected directly in the resources (i.e., computing power, memory) needed by the IoT module, which should carry out the classification task.

## 4. Results

The assessment of the proposed IoT-based acoustic classification system was done on a test set with 643 recordings, which were held out from the acoustic models’ training set. The offline evaluation was carried out for different acoustic models prepared during the training phase. Classification Accuracy (*Acc*) was applied as a metric for the evaluation. It is defined as:

(1) $$Acc\left(\%\right)=\frac{H}{N}\cdot 100$$

where *H* is the number of correctly recognized bee activity events, and *N* is the number of all activity events in the particular test set. The test set results were also evaluated using Precision (P), Recall (R) and F1-score as metrics.

The first part of the evaluation was devoted to the analysis of which types of acoustic models were more suitable for bee swarm activity acoustic classification. Three different types of acoustic models with MFCC features were compared in this evaluation. The HMM acoustic models were represented with 1-state and 15-state models, and the standard GMM models were the third ones in the set. The number of mixtures per state usually correlates directly with the computational complexity in the case of the bee swarm activity classification. Therefore, acoustic models were evaluated with 2, 8 and 32 Gaussian PDF mixtures per state. Thus, the acoustic models’ set included one simple, one medium, and one high, complexity representative. The first one could be applied if computational resources would be low, which can occur frequently in the case of IoT devices installed directly in the beehive, which have only an embedded processor available. In this case, the IoT device could be used to monitor the bee colony independently, without the availability of any high data rate mobile network which would usually be needed in the case if classification would be carried out in real time in the cloud, and only the audio capturing would be performed locally on the IoT module in the beehive. The IoT module could also be equipped with a GPU-embedded module, which would increase its computational power for the classification task. The most complex acoustic models with 32 Gaussian mixtures per state were expected to achieve the best result, but with the drawback of higher computational costs. This set of acoustic models is most suitable for server-side operations in a cloud, where the role of an input client goes to an IoT audio capture sensor module, and the function output provides farming monitoring services with the information about beehive conditions. The results of normal versus swarm bee activity classification for various acoustic models are presented in Table 1 and Table 2.

The less complex bee activity acoustic classification system achieved approx. 60% accuracy, distinguishing between normal and swarm conditions. This part of the evaluation shows that the GMM acoustic model provided the best classification accuracy of 60.50%, while 1-state HMM acoustic models performed worse, with 58.94% accuracy. The classification results for acoustic models with eight Gaussian mixtures per state are presented in the second row in Table 1 and Table 2. Here, the best classification accuracy of 75.43% and 0.86 F1-score was achieved with the 15-state HMM acoustic models. The increased number of acoustic models’ parameters contributed to the statistically significant improvement of its classification. This time, the worst result belonged to the GMM acoustic models, which achieved 71.54% classification accuracy and a 0.83 F1-score, which also presented the largest performance gap between the HMM and GMM types of acoustic models. The possible cause of this accuracy change could originate from the acoustic modeling approach. An additional increase of acoustic models’ parameters to 32 Gaussian mixtures, and in consequence, also the increase of complexity, improved the classification accuracy, although to a smaller extent than in the previous scenario. The highest bee activity classification accuracy of 82.27% was again achieved with the 15-state HMM acoustic models. The precision was 0.89, recall 0.92 and F1-score 0.90. This presents the best overall classification results. The 1-state HMM and GMM acoustic models performed less successfully, with classification accuracies of 80.40% and 79.78%, respectively. The presented results show that all three acoustic models type can classify bee swarm activity successfully, with the 15-state HMM acoustic models being the most suitable ones. A possible explanation could be that the 15-state HMM acoustic models were able to model the temporal variations better in the captured audio signal.

The second part of the evaluation was focused on the question of how feature extraction influences the classification. The cepstral mean normalization was added to the MFCC feature vectors and LPC features were compared with both MFCC methods. The experiments were repeated only with the HMM acoustic models with fifteen states, as this acoustic model succeeded in achieving the best result in the previous part of the evaluation. The results for the evaluation of feature vectors are presented in Table 3 and Table 4. Three different acoustic model complexities were included in the evaluation.

With low complexity acoustic models, the MFCC features achieved 59.88% classification accuracy and F1-score 0.75. MFCC features with cepstral mean normalization achieved 60.81% accuracy and F1-score 0.76. With the medium and high complexity acoustic models, the situation was vice versa, with 75.43% and 82.27% classification accuracy without cepstral mean normalization, and 74.81% and 81.96% with it, respectively. An increased number of Gaussian mixtures reduced the accuracy performance gap between both feature extraction types, with a difference in results which is not statistically significant. This indicated that cepstral mean normalization did not introduce any additional benefit for the experimental setup. One possible explanation could be that the spectral characteristics of an acoustic input signal do not differ a lot between training and test material, although both of them were recorded over a large time interval from a single beehive. In the case when a higher number of locations would be included in the audio data set, the cepstral mean normalization would probably be of benefit. The LPC feature extraction method performed slightly worse when compared to MFCC feature extraction. The LPC classification accuracy with 2, 8 and 32 Gaussian PDF mixtures was 59.25%, 71.07% and 80.40%, respectively. The LPC feature extraction produced slightly higher precision (i.e., misdetected swarm activity) than MFCC and MFCC_CMN, but at the cost of lowering the F1-score because of reduced recall.

The same configuration of acoustic models and the MFCC feature extraction procedure was used to analyze the computer power and memory needed to carry out the evaluation (Table 5). The classification Real-Time Factor (RTF) was measured on a single core of the Intel Xeon CPU running at 2.2 GHz, which was used as a computation server for the experiments.

The memory size needed for the acoustic models increased proportionally with the number of mixtures of Gaussian probability density functions per state. The size of the smallest model was 25 kB, and the size of the model with 32 mixtures was 355 kB. The real-time factor measured on the test set was between 0.003 and 0.009. The fast RTF was mainly the result of the topology applied for the classification. The computation ratio between feature extraction and classification procedure depended strongly upon the number of mixtures of Gaussian probability density functions which defined the needed time for classification.

On the other hand, the time needed for feature extraction was constant, with RTF of 0.002. The classification performance analysis indicated that the proposed system could also be used in an on-line mode of operation.

The third part of the evaluation addressed the influence of background noise and denoising procedure on bee activity acoustic classification. The evaluation on the noisy test set was carried out with the MFCC feature extraction and 15-state HMM acoustic models with eight Gaussian PDF mixtures per state. The results are given in Table 6.

The added urban background noise had a significant impact on bee acoustic classification results. Already, the low level of noise degraded the accuracy to 70.76% and the F1-score to 0.83. In the case when a high level of noise was added, the classification degraded severely. The accuracy was 51.17% and the F1-score 0.68. The denoising with spectral subtraction could reduce the degradation in a case of low-level noise, where the accuracy reduced to 71.85%. With the high-level noise, the denoising further degraded the results, achieving an accuracy of 48.83% and an F1-score of 0.66. A possible cause could be the spectral similarity of the input signal and noise at the high level of degradation. The classification results with background noise indicate that the proposed system can, to some degree, handle the background noise.

**Figure 6:**
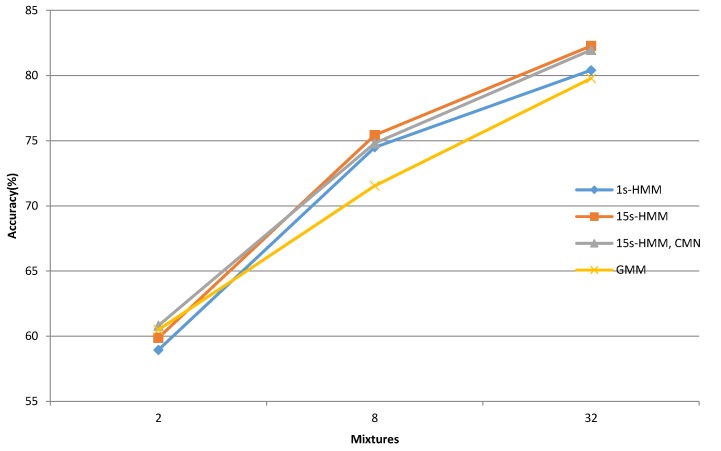
Bee activity acoustic classification accuracy comparison.

The overall comparison of bee activity acoustic classification accuracy is presented in Figure 6.

In general, it can be observed that the proposed IoT-based system copes successfully with the task. If IoT architecture should be considered, the HMM models of high or middle complexity with MFCC features and without cepstral mean normalization are the most suitable choice. Different other machine learning approaches [31], such as CNN, can be also used for animal acoustic classification or analysis [32]. The CNN-based system can provide high accuracy when large amounts of acoustic training material are available. Compared to HMM/GMM-based solutions, CNN also demands higher computational power, which can be an important factor if IoT solutions are taken into account.

## 5. Conclusions

IoT technologies have evolved rapidly over the last decade, and are moving successfully to agriculture, where beekeeping presents one of the important disciplines. The proposed IoT-based acoustic classification system is capable of monitoring bee swarm activity from the captured input audio signal. The system is based on MFCC and LPC feature extraction. Three different types of acoustic models were included in the setup. The best performance was achieved with the system based on HMM acoustic models and MFCC features. This could be important in the case if the proposed system would be part of a larger intelligent ambiance system encompassing various IoT modules. The difference between the GMM and HMM approach in the case of medium topology needs to be analyzed further. The evaluation shows promising results. If some limitations connected with the setup would be taken into account, the system could be used in an IoT environment. The future work will be oriented on the topic of how to improve the accuracy at the level of additional classification categories.

## Figures and Tables

**Table 1:** Accuracy results for bee acoustic classification with MFCC features and three different acoustic models types.

Probability Density Functions per State	Accuracy (%)		
	1-State HMM	15-State HMM	GMM
2 Gaussian mixtures	58.94	59.88	60.50
8 Gaussian mixtures	74.49	75.43	71.54
32 Gaussian mixtures	80.40	82.27	79.78

**Table 2:** Precision, Recall and F1-score for bee acoustic classification with MFCC features and three different acoustic models types.

Probability Density Functions per State	1-State HMM			15-State HMM			GMM		
	P	R	F1	P	R	F1	P	R	F1
2 Gaussian mixtures	0.72	0.76	0.74	0.72	0.78	0.75	0.77	0.74	0.75
8 Gaussian mixtures	0.82	0.89	0.85	0.85	0.87	0.86	0.86	0.81	0.83
32 Gaussian mixtures	0.85	0.93	0.89	0.89	0.92	0.90	0.89	0.88	0.89

**Table 3:** Accuracy results for bee acoustic classification without (MFCC) and with cepstral mean normalization (MFCC_CMN) and with the Linear Predictive Coding (LPC) features.

Probability Density Functions per State	MFCC	MFCC_CMN	LPC
2 Gaussian mixtures	59.88	60.81	59.25
8 Gaussian mixtures	75.43	74.81	71.07
32 Gaussian mixtures	82.27	81.96	80.40

**Table 4:** Precision, Recall and F1-score for bee acoustic classification without (MFCC) and with cepstral mean normalization (MFCC_CMN) and with the LPC features.

Probability Density Functions per State	MFCC			MFCC_CMN			LPC		
	P	R	F1	P	R	F1	P	R	F1
2 Gaussian mixtures	0.72	0.78	0.75	0.74	0.77	0.76	0.79	0.70	0.74
8 Gaussian mixtures	0.85	0.87	0.86	0.84	0.87	0.86	0.88	0.79	0.83
32 Gaussian mixtures	0.89	0.92	0.90	0.89	0.91	0.90	0.90	0.88	0.89

**Table 5:** Real-Time Factor (RTF) ratio between Feature Extraction (FE) and Classification (CA) and the acoustic models’ memory sizes.

Probability Density Functions per State	Memory (kB)	RTF	FE:CA Ratio
2 Gaussian mixtures	25	0.003	0.75:0.25
8 Gaussian mixtures	91	0.004	0.54:0.46
32 Gaussian mixtures	355	0.009	0.26:0.74

**Table 6:** Accuracy, precision, recall and F1-score for bee acoustic classification with background noise and with the denoising procedure.

Noisy Test Set	MFCC				Denoising			
	Acc	P	R	F1	Acc	P	R	F1
Original recording	75.43	0.85	0.87	0.86	75.58	0.85	0.88	0.86
Low noise	70.76	0.81	0.85	0.83	71.85	0.82	0.85	0.84
High noise	51.17	0.63	0.73	0.68	48.83	0.62	0.70	0.66
